# Influencing factors of cardiorespiratory fitness in allogeneic stem cell transplant candidates prior to transplantation

**DOI:** 10.1007/s00520-020-05485-y

**Published:** 2020-05-04

**Authors:** Matthias Limbach, Rea Kuehl, Peter Dreger, Thomas Luft, Friederike Rosenberger, Nikolaus Kleindienst, Birgit Friedmann-Bette, Andrea Bondong, Martin Bohus, Joachim Wiskemann

**Affiliations:** 1 Department of Medical Oncology, National Center for Tumor Diseases (NCT), Heidelberg University Hospital, Im Neuenheimer Feld 460, 69120 Heidelberg, Germany; 2 Department of Medicine V, Heidelberg University Hospital, Heidelberg, Germany; 3 Central Institute of Mental Health, Mannheim, Germany; 4 Internal Medicine VII (Sports Medicine), Heidelberg University Hospital, Heidelberg, Germany

**Keywords:** Leukemia, Lymphoma, Risk management, Exercise, Oncology

## Abstract

**Purpose:**

Cardiorespiratory fitness (CRF) seems to be prognostic prior to allogeneic stem cell transplantation (allo-HSCT). Influencing factors of CRF in allo-HSCT candidates have not been studied so far. Aim was to identify potentially influencing factors on CRF.

**Methods:**

To assess CRF, a maximal cardiopulmonary exercise test (CPET) was performed on average 2.6 ± 7.2 days prior to admission. A regression analysis was conducted, with the following predictors: gender, age, body mass index (BMI), time between last therapy and allo-HSCT (t_Therapies), number of cardiotoxic therapies (n_Cardiotox), number of transplantations (n_Transplantations), comorbidity index (HCT-CI), hemoglobin level of the last 3 months (area under the curve), and physical activity.

**Results:**

A total of 194 patients performed a CPET. VO_2peak_ was significantly reduced compared with reference data. In total, VO_2peak_ was 21.4 ml/min/kg (− 27.5%, *p* < 0.05). Men showed a significant larger percentage difference from reference value (− 29.1%, *p* < 0.05) than women (− 24.4%). VO_2peak_ was significantly (*p* < 0.05) influenced by age (β = − 0.11), female gender (β = − 3.01), BMI (β = − 0.44), n_Cardiotox (β = − 0.73), hemoglobin level (β = 0.56), and physical activity prior to diagnosis (β = 0.10).

**Conclusions:**

Our study demonstrates a decreased CRF indicating the potential need of prehabilitative exercise. We revealed some influencing factors on CRF. Those patients could benefit the most from exercise.

## Introduction

The number of performed allogeneic hematopoietic stem cell transplantations (allo-HSCT) worldwide has increased over the past decades [1]. This development is related to the extended donor availability and improved transplant techniques such as reduced intensity conditioning or a better usage of the immunotherapeutic graft-versus-leukemia effect, as well as enhanced supportive measures [2]. As a consequence, mortality rates of allo-HSCT are decreasing constantly, however, remain on a considerable high level [2]. A recent retrospective cohort study demonstrated a decline for non-relapse mortality from 41 to 26% comparing the years 1993–1997 and 2003–2007 [3]. Therefore, an accurate risk-benefit stratification is still vital for decision-making in allo-HCT candidates.

Several assessment tools are available for calculating individual patient risk and to define suitable candidates for allo-HSCT like the comorbidity index (HCT-CI), the Karnofsky performance status (KPS), or the European Group for Blood and Marrow Transplantation (EBMT) risk score [4–6]. Recently, measurements of cardiac and pulmonary function were introduced to determine patients’ physical function and possess prognostic information prior to transplantation [7, 8]. It could be demonstrated that decreased cardio respiratory fitness (CRF) values are associated with a lower health-related quality of life, a higher symptom burden, and a higher risk of mortality in patients undergoing HSCT [8]. This has also been shown in other cancer populations [9, 10].

Since CRF could be modified by exercise intervention approaches, there is a certain interest to identify factors explaining lower CRF value prior to HSCT. However, the pre-treatment situation in HSCT patients is quite complex, and it is still uncertain on what affects the physical performance prior to HSCT. Initial approaches emphasized that the amount of prior chemotherapies or months of received chemotherapy prior to HSCT might impact CRF [8], but comprehensive data analyses are still missing. Therefore, we aimed to evaluate CRF and its influencing factors in patients immediately prior to allo-HSCT.

## Patients and methods

### Setting and patients

We analyzed baseline data from *n* = 256 allo-HSCT patients from the study Physical Exercise Training versus Relaxation in Allogeneic stem cell transplantation (PETRA), a large ongoing two-arm randomized controlled trial (RCT) of exercise intervention. All patients, scheduled for an allo-HSCT at the Heidelberg University Hospital, age ≥ 18 years and able to understand and follow the study protocol were eligible to be enrolled in the PETRA study. Exclusion criteria were inability to walk or stand, unstable bone lesions, severe neurological deficiencies, severe cardiac or cardiovascular diseases, and severe pulmonary global insufficiency. Cross-sectional data were obtained prior to admission for allo-HSCT. The study has been approved by the ethics committees of the Ethics Committee II of the University of Mannheim (number 2009–349 N-MA) and the Ethics Committee of the University of Heidelberg (number S-021/2011), and is registered at ClinicalTrials.gov (NCT01374399). All patients provide written informed consent.

### Determination of CRF

To assess baseline CRF of the participants, a cardiopulmonary exercise test (CPET) was performed prior to admission. The CPET was conducted on an electronically braked bicycle ergometer (ergoselect 100, Bitz, Germany and Corival, Lode B.V. Medical Technology, Groningen, Netherlands). Gas exchange was measured using a breath by breath system (Ergostik, Geratherm Respiratory, Bad Kissingen, Germany), which was calibrated according to the instructions of the manufacturer before each test. A stepwise incremental protocol was applied, which started at 50 W, and work rate was increased every 2 min by 25 W until voluntary exhaustion or occurrence of medical reasons. Cadence was kept constant between 60 and 70 rpm. A 12-lead electrocardiogram continuously monitored and blood pressure was measured every 2 min during the test. For analysis, we determined VO_2peak_ and the VO_2peak_ in relation to body weight (ml/min/kg) referring to the ATS/ACCP recommendations [11]. VO_2peak_ and heart rate were considered the highest 30s average value during or immediately post-termination CPET. We used the following criteria to determine patients’ exhaustion: respiratory exchange ratio (RER) > 1.1 or HR > 85% of age predicted maximum.

### Potentially influencing factors for CRF

Additionally, we investigated potentially influencing factors for CRF. Patients were asked to self-report the level of physical activity (walking, cycling, and other sports) in a typical week in the year before diagnosis with a proved questionnaire [12]. The degree of physical activity was operationalized with metabolic equivalent of task (MET). MET hours per week (MET × h/week) were calculated by summing the average hours per week spent in walking, cycling, or other sports. The following clinical data prior to allo-HSCT were extracted from the medical records as potentially influencing factors: number of cardiotoxic therapies (n_Cardiotox), number of transplantations (allogeneic and autologous, n_Transplantations), months between last therapy to allo-HSCT (t_Therapies), KPS, remission status, the hematopoietic cell transplantation-specific comorbidity index (HCT-CI), primary hematological malignancies, LVEF (left ventricular ejection fraction), hemoglobin levels, age, gender, and body mass index (BMI). BMI was calculated as body weight divided by square of height in meters. Hemoglobin (g/dl) level was measured on the day of CPET assessment. Further, we calculated area under the curve values for hemoglobin levels over the last 3 months prior to allo-HSCT (Hemoglobin_auc). The HCT-CI was determined according to guidelines and separated into low to intermediate risk group (< 3) and high risk group (≥ 3) [13].

### Statistical analyses

Baseline clinical and demographic data were reported by descriptive analysis and are reported as mean ± SD and range. Differences between measured values of VO_2peak_ and reference values were reported as percent differences. The observed values were compared with reference values by using paired sample *t* test. Expected individual VO_2peak_ values were calculated by Koch [14] for healthy people of considering age, gender, and BMI distribution. Hemoglobin level over the last 3 months was calculated as area under the curve score using the trapezoidal rule [15]. To identify determinants of CRF prior to allo-HSCT, a multivariate regression analysis was conducted. The following independent variables were entered into the regression model simultaneously: age, gender, BMI, n_Transplantations, n_Cardiotox, t_Therapies, HCT-CI, Hemoglobin_auc, and physical activity. We chose VO_2peak_ as representative value for CRF and therefore as the dependent variable. In the second regression model, the deviation VO_2peak_ from healthy reference was used as the dependent variable. The level of statistical significance was set at *p* < 0.05. Data were analyzed using IBM SPSS v. 22.

## Results

**Fig. 1:**
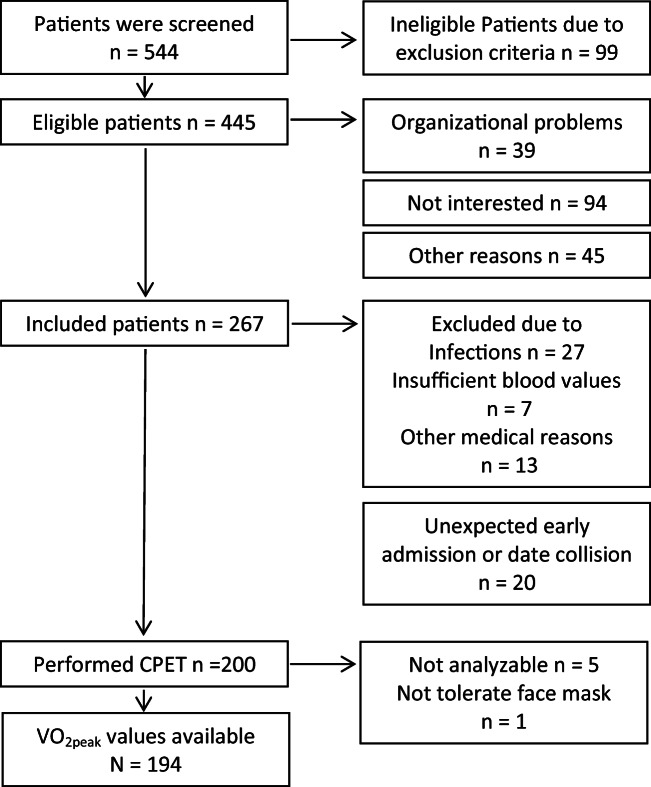
Patient flow chart showing the numbers of patients who were available for cardiopulmonary exercise test (CPET)

Five hundred forty-four patients were screened and *n* = 99 were ineligible due to exclusion criteria (18.2%). From *n* = 445 (81.8%) eligible patients, *n* = 178 (40%) did not participate. Reasons were *n* = 94 (52.8%) not interested, *n* = 39 (21.9%) due to organizational problems (postponement, no exercise testing capacity available due short notice admission to allo-HSCT), and *n* = 45 (25.3%) because of other reasons. Two hundred sixty-seven patients were included of whom *n* = 200 participated in CPET. Major reasons for not being able to perform CPET were infections *n* = 27 (10.1%), insufficient blood values *n* = 7 (2.6%), and other medical reasons *n* = 13 (4.9%). Twenty patients (7.5%) did not participate due to unexpected early inpatient admission or unpredictable date collision. Five of *n* = 200 VO_2peak_ values were not analyzable. One patient did not tolerate the face mask (Fig. 1). No serious adverse events occurred.

Clinical data and demographics of study participants who performed CPET are described in Table 1. Patients had a mean age of 54.1 ± 11.5 years. Main diagnosis was acute leukemia (AML, 29.9%). They were on average 36.6 ± 47.5 months after main diagnosis. Mean hemoglobin at the day of CPET testing was 11.7 ± 1.8 (g/dl) and hemoglobin level during the last 3 months (Hemoglobin_auc) was 11.4 ± 1.9 (g/dl). On average, the patients performed the CPET 2.6 ± 7.2 days prior to admission. There is a significant association between n_Transplantations and n_Cardiotox (*r* = 0.502, *p* = <0.001). The analysis of number of transplantations in the patients history showed that *n* = 47 (24.2%) patients received one or more previous transplantations.

**Table 1:** Baseline descriptive data

	Number	Mean	SD	Range
Age (year) at diagnosis	194	54.1	11.5	18–75
Gender, *n* (%)	194 (100)	-	-	-
Males	130 (67)			
Females	64 (33)			
BMI (kg/m^2^)	192	26.5	5.1	17–57
Diagnosis, *n* (%)	194 (100)	-	-	-
AML	58 (29.9)			
ALL	10 (5.2)			
CLL	39 (20.3)			
MM	19 (9.8)			
CML/MPS	17 (8.7)			
MDS	16 (8.2)			
Other lymphoma	32 (16.3)			
Other	3 (1.6)			
Time from diagnosis to allo-HSCT (month)	194	36.6	47.5	0–328
Time between exercise test and admission allo-HSCT (days)	194	2.6	7.2	0–47
HCT-CI (*n*)	184	0.9	1.5	0–8
< 3	158			
≥ 3	26			
Missing	10			
KPS (*n*)	190	92.7	6.5	100–70
< 90	19			
≥ 90	171			
Missing	4			
Hemoglobin day of CPET testing (g/dl)	194	11.7	1.8	6.8–17.0
LVEF (*n*)	181	-	-	-
> 55%	163			
45–54%	14			
30–44%	4			
< 30%	0			
Missing	13			
Remission status prior to allo-HCT (*n*)	193	-	-	-
CR	70			
PR	73			
No CR	46			
Unknown	4			
Missing	1			
MET (h/week)	194	7.6	14.9	0–99.5
Smokers (*n*)	185			
Current	8			
Ever	62			
Year before diagnosis	27			
Never	88			
Missing	7			
VO2peak_rel (ml/kg/min)	194	21.4	5.7	8–41
VO2peak (l/min)	194	1.71	0.5	0.7–3.4
Time since last therapy (month)	194	2.3	4.4	0–54
Hemoglobin last 3 months (g/dl)	192	11.4	1.9	6.5–15.7
Missing	2			
Amount of cardio toxic agents	194	1.5	1.4	0–9
Number of previous transplantations	194	0.3	0.6	0–3

*N*, number of patients; *BMI*, body mass index; *HCT-CI*, comorbidity index by Sorror; *KPS*, Karnofsky performance status; *LVEF*, left ventricular ejection fraction; *allo-HCT*, allogeneic stem cell transplantation; *CR*, complete remission; *PR*, partial remission; *AML*, acute myeloid leukemia; *ALL*, acute lymphocytic leukemia; *MM*, multiple myeloma; *CML*, chronic myeloid leukemia; *MPS*, myeloproliferative syndrome; *MDS*, myelodysplastic syndrome; *MET*, metabolic equivalent of task

### CRF in relation to normal values

**Fig. 2:**
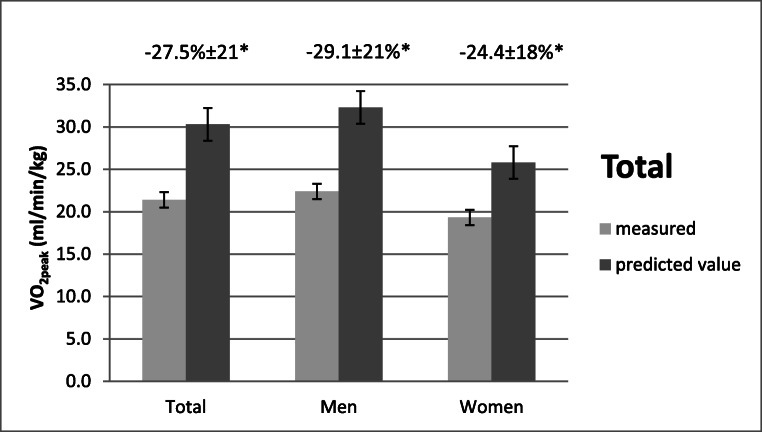
Comparison of measured VO_2peak_ values with gender- and age-matched healthy reference VO_2peak_ values in total, men and women. * = significant difference to measured VO_2peak_ and reference values *p* < 0.005

VO_2peak_ values were available in 194 patients. All descriptive data of predictors and VO_2peak_ are depicted in Table 1. Figure 2 shows the deviation of measured VO_2peak_ values and the healthy reference values of CRF.

For the overall sample, VO_2peak_ was on average 21.4 ml/min/kg, with a value of 22.4 ± 5.9 ml/min/kg for men and 19.3 ± 4.7 ml/min/kg for women. Figure 2 presents the comparison with age- and gender-matched reference values [14]. For the total sample, it was apparent that the VO_2peak_ values were significantly reduced (− 27.5 ± 20.8%, *p* < 0.001). The deviation of VO_2peak_ from gender- and age- matched healthy reference values was slightly larger in men (− 29.1 ± 21.6%, *p* < 0.001) than in women (− 24.4 ± 18.1%, *p* < 0.001), with no significant difference between the two groups (− 4.7%, *p* = 0.146).

Hemoglobin at the day of CPET was higher in men (11.9 ± 1.8 g/dl) than in women (11.3 ± 1.5 g/dl), however there was no statistically difference between men and women (men vs woman 4.7%, *p* = 0.766). Hemoglobin values were below the normal range.

Table 2 shows the deviation of VO_2peak_ from healthy reference values, the measured VO_2peak_ values, the amount of cardio toxic agents, and the activity level per week (MET) in the year before diagnosis depending on different age groups. The age group between 46 and 55 years demonstrated the largest deviation from healthy reference values (− 36.6% ± 16.7), whereas the eldest (60–75 years) illustrated the smallest deviation (− 11.8% ± 15.9). The amount of cardio toxic agents was highest in the youngest age group (1.9 ± 1.3) with small differences to the other age groups, whereas the oldest participants notably received the least amount (0.9 ± 1.9).

**Table 2:** Comparison of measured VO_2peak_ (ml/kg/min) values and the healthy gender-age-matched reference VO_2peak_ values in total, n_Cardiotox, MET h/week

Age group (years)	Number	Deviation VO_2peak_ from healthy reference (%) (mean ± SD)	VO_2peak_ (ml/kg/min) (mean ± SD)	n_Cardiotox (mean ± SD)	MET h/week (mean ± SD)
18–35	18	− 25.1 ± 15.7	24.7 ± 6.3	1.9 ± 1.3	3.3 ± 5.4 (*n* = 6)
36–45	13	− 31.7 ± 18.7	23.9 ± 7.4	1.5 ± 1.4	8.1 ± 13.9 (*n* = 4)
46–55	59	− 36.6 ± 16.7	20.2 ± 5.6	1.7 ± 1.4	6.1 ± 13.1(*n* = 14)
56–65	80	− 25.4 ± 22.9	21.7 ± 5.5	1.4 ± 1.2	9.2 ± 17.5 (*n* = 27)
66–75	24	− 11.8 ± 15.9	19.1 ± 4.1	0.9 ± 1.9	9.2 ± 14.8 (*n* = 9)

*N*, number of patients; *MET*, metabolic equivalent of task; *HCT-CI*, comorbidity index by Sorror

### Factors explaining CRF

A total of 183 patients could be included in the multiple regression analysis. The analysis reveals age, female gender, BMI, n_Cardiotox, Hemoglobin_auc, and physical activity prior to diagnosis as predictors with a significant influence on VO_2peak_, whereas the HCT-CI, the amount of transplantations (n_Transplantations), and t_Therapies had no significant impact. The *R*^2^ was 40% (Table 3).

**Table 3:** Multiple regression of determinants VO_2peak_ (ml/min/kg) *R*^2^ = 40%, *n* = 183

	β	*p*	95% CI
Gender	− 3.01	0.000	− 4.44; − 1.58
Age	− 0.11	0.003	− 0.17; 0.05
BMI	− 0.44	0.000	− 0.58; − 0.31
t_Therapies	0.11	0.146	− 0.04; 0.27
n_Cardiotox	− 0.73	0.015	− 1.33; − 0.11
HCT-CI	− 0.34	0.138	− 0.79; 0.11
Hemoglobin_auc	0.56	0.004	0.18; 0.93
n_Transplantations	0.45	0.658	− 0.89; 1.78
Physical activity	0.10	0.000	0.05; − 0.14

*BMI*, body mass index; *HCT-CI*, comorbidity index by Sorror; *CI*, confidence interval

### Factors explaining deviation of CRF from normative values

Regression analysis for the deviation of VO_2peak_ from healthy reference values shows that age, gender, BMI, Hemoglobin_auc, and physical activity prior to diagnosis are significant determinants. The explaining variance is 26% (Table 4).

**Table 4:** Multiple regression of determinants deviation VO_2peak_ (ml/min/kg) from healthy reference (%) *R*^2^ = 26%, *n* = 183

	β	*p*	95% CI
Gender	7.58	0.001	1.72; 13.43
Age	0.043	0.000	0.19; 0.67
BMI	− 1.12	0.000	− 1.68; − 0.57
t_Therapies	0.13	0.673	− 0.48; 0.75
n_Cardiotox	− 2.46	0.047	− 4.89; − 0.36
HCT-CI	− 0.30	0.750	− 2.15; 1.55
Hemoglobin_auc	1.99	0.001	0.46; 3.52
n_Transplantations	1.56	0.575	− 3.91; 7.02
Physical activity	0.41	0.000	0.22; 0.60

*BMI*, body mass index; *HCT-CI*, comorbidity index by Sorror; *CI*, confidence interval

## Discussion

Our study demonstrates decreased CRF in allo-HSCT candidates prior to transplantation. Results indicate that patients at higher age, of female gender, with low hemoglobin values, who received cardio toxic treatment prior to allo-HSCT, and have a low physical activity are of risk for lower CRF. Considering gender- and age-matched healthy VO_2peak_ reference values, men as well as patients with a younger age have a higher risk for lower CRF. A higher BMI and lower physical activity was a significant predictor for low CRF in both regression models.

Reduced physical performance levels prior to HSCT has been found in various studies [7, 8, 16, 17]. However, comprehensive data and analyses procedures are missing to identify patients at risk and to assess the clinical significance of reduced CRF. In comparison with reference values of healthy adults, the VO_2peak_ of our sample was significantly reduced by − 27.5% which was quite similar to other studies in the field [7, 8]. This is further in accordance with findings of impaired respiratory and skeletal muscle strength as well as reduced submaximal exercise capacity in patients prior to allo-HSCT [16–18]. In comparison with other severely impacted and treated patient groups like pancreatic cancer patients, the deviation from the healthy reference group was higher [19], whereas breast cancer patients at the same age interestingly showed lower VO_2peak_ values [20].

Since reduced CRF level is associated with poor prognosis after HSCT [8], but on the other hand can be positively modified by exercise therapy [21, 22], it is of clinical relevance to detect patient-related predictors in order to identify low CRF patients and to treat them as early as possible in accordance with the prehabilitation approach [23]. Regarding patient-related predictors, we were able to show that the amount of chemotherapies with cardio toxic ingredients received prior to allo-HSCT is a predictor of low CRF. In light of the fact that 24.2% of our population received one or more transplantation prior to allo-HSCT, which frequently include cardio toxic agents and affect cardiac function [24, 25] such an observation seems to be plausible [26]. Interestingly, although the number of received cardio toxic treatments could explain variation in CRF, the majority of our population (90%) showed good LVEF values indicating no major structural impact on the heart muscle. The detection of heart damage after exposure to potentially cardio toxic chemotherapeutic agents is complex and requires various assessment procedures [27] and was not performed within this study. Nevertheless, an explanation for this finding could be that assessments without exercise-induced cardiovascular stress, like a resting echocardiography, are not sensitive enough to detect preliminary heart damage [7]. In addition, it is conceivable that the toxic components of chemotherapies change intramuscular capillary density and mitochondrial content, and therefore reduce the oxygen extraction from the blood to the muscles, leading to reduced VO_2peak_ values [28]. Causes of muscle dysfunction in cancer patients are complex and very little is known about adaptions at the muscle structural level [29]. However, improved muscle strength seems to have an impact on peak work rate in a CPET, but not on VO_2peak_ in pancreatic cancer patients. This could also be explained by peripheral intramuscular changes and increase in cross-sectional areas of muscle fibers [30]_._ Further interpretations are difficult, because measures of muscle strength were not part of our present analysis. However, our data show that it may be worthwhile to carry out further investigations including measures of muscle strength.

Not unexpected, we showed that low levels of hemoglobin for at least 3 months prior to allo-HSCT were another predictive factor for low CRF. Reduced hemoglobin levels as a result of reduced bone marrow capacity due to the disease itself or chemotherapy treatment could limit oxygen delivery to exercising muscles and could affect CRF [31]. Low hemoglobin is associated with a lower quality of life and functional capacity [32] and is a prognostic factor for overall survival and disease-free survival in several hematological malignancies [33].

Further, gender had a significant impact on CRF. Women had lower VO_2peak_ values than men, which is a well-known gender-related finding [34]. However, men showed slightly greater, but non-significant deviations, from heathy reference values than women (− 29.1% vs − 24.4%). This is not in line with findings of other comparable studies [8], but was similarly reported in solid cancer patients [19].

Regarding age, we found that patients between 46 and 55 years revealed reduced CRF, whereas the oldest age group (66–75 years) had the lowest deviation from healthy norm values. An explanation for this finding could be related to the fact that older patient groups mostly receive dose-reduced therapies [35] compared with younger patients and therefore experience lower grades of myelosuppression and hematotoxicity. Otherwise, older cancer patients might be at increased risk of chemotherapy-related cardiotoxicity due to physiologic changes of several organ systems [36] or reduced tolerance of drug toxicity due to lower lean mass [37]. However, age- and treatment-associated variables are independently explaining variation in CRF and therefore seem to be both important for identifying patients with low CRF.

As expected, our data in both regression models show that physical inactivity prior to allo-HCT affects CRF negatively. In observational studies, it could be shown that a higher level of physical activity has a positive influence on the treatment process [38], which may additionally promote CRF even before allo-HSCT. Additionally, our models show that patients with higher BMI had a significantly lower CRF. This finding could be partially explained due to the fact that body weight is part of both formulas (BMI and VO_2peak_/ml/min/kg, respectively). Furthermore, the increased body mass also requires more oxygen during exercise, leading to reduced values. In allo-HSCT patients, both under- and overweight were associated with increased risk for complications and lower overall survival [39]. However, drawing conclusions from BMI regarding the association to CRF is difficult because the BMI gives no detailed information on the distribution of fat mass and muscle mass. In the field of allo-HSCT, for example, pre-transplant weight loss has been shown to be a prognostic variable [40].

Based on our analyses, we were able to identify and characterize patients with low CRF prior to allo-HSCT. Past studies have shown that reduced CRF predicts symptom severity and mortality in patients undergoing allo-HSCT [8, 18]. Consequently, we recommend based on our data a targeted exercise program to create the best possible condition for allo-HSCT. This would lead to a prehabilitation approach and is in line with current recommendations from the literature, “getting fit for allogeneic hematopoietic cell transplantation” [41]. Beneficial examples for prehabilitation in cancer patients exist [42, 43], but are totally understudied in hematological patients prior to allo-HSCT. Nevertheless, exercises have the potential to improve CRF in patients preparing to undergo HCT [23], which is potentially crucial for survival and for reducing treatment-related side effects [44, 45]. Our data reinforce timely supportive care with exercise and identify needy subgroups in allo-HSCT candidates. For a better planning of exercise therapies, patients with low physical activity level, who are middle-aged, who have a high BMI, who had received cardio toxic agents, and who have chronic low hemoglobin levels should be the targeted population.

Our investigation has several limitations. The retrospective analysis was based on data of a randomized exercise intervention trial and therefore was not stratified and designed to evaluate baseline characteristics concerning potentially influencing factors with regard to CRF in patients prior to allo-HSCT. This might have also led to a preselection of a population with a potential affinity to exercise. Further, clinical data prior to allo-HSCT was extracted from the medical records which led to incomplete information.

This investigation also has several strengths. To our knowledge, the study sample of PETRA participant is currently the largest providing comprehensive cardiorespiratory fitness data applying gold standard assessment. Due to the large sample size, it was possible to identify influencing factors, which allow a more profound understanding of patients’ performance status and its influencing parameters prior to allo-HSCT.

In conclusion, our findings demonstrated considerable reduction of CRF in patients immediately prior to allo-HSCT. We found that high BMI, low physical activity, amount of cardio toxic agents, and low hemoglobin level negatively impact CRF prior to allo-HSCT. Since CRF is proposed to be an independent risk factor for transplant outcome, strategies to modify CRF prior to allo-HSCT might be worthwhile to follow. Therefore, structured individualized aerobic exercise intervention programs should be recommended for increasing CRF prior to allo-HSCT. Based on our findings, describing risk factors for low CRF prior to allo-HSCT could lead to a targeted prehabilitative exercise intervention approach identifying those patients with highest needs.

## Data Availability

Not applicable
